# Long-term effects of mentalization-based treatment for psychotic disorder: a 5-year follow-up of a multi-center, randomized-controlled trial

**DOI:** 10.1017/S0033291725102821

**Published:** 2026-01-09

**Authors:** Jonas Gijs Weijers, Martin Debbané, Coriene ten Kate, Fleur van Kaam, Remco de Winter, Elisabeth Eurelings-Bontekoe, Jean-Paul Selten

**Affiliations:** 1 Mental Health and Neuroscience, Maastricht University Faculty of Health, Medicine and Life Sciences: Maastricht, Maastricht, Netherlands; 2 Clinical Psychology, University of Geneva Faculty of Psychology and Education: Universite de Geneve F, Geneva, Switzerland; 3 MBT team, GGZ Rivierduinen, Leiden, Netherlands; 4 Clinical Psychology, Leiden University: Universiteit Leiden, Leiden, Netherlands

**Keywords:** flexible assertive community treatment, long-term outcomes, mentalization-based treatment, mentalizing capacity, psychosis, social functioning

## Abstract

**Background:**

The results of a previous randomized trial showed that mentalization-based treatment for psychotic disorder (MBTp) was associated with greater improvement than treatment as usual (TAU) in social functioning up to 6 months after treatment. The purpose of the present study is to examine the effect after 5 years.

**Methods:**

The researchers tried to find all patients who had participated in the trial (n = 84) and to assess, blind to previous treatment status, their social functioning and mentalizing capacity. Social functioning was measured using the Social Functioning Scale, mentalizing using the Social Cognition and Object Relations Scale and the Hinting Task.

**Results:**

Twenty-three MBTp patients and 23 TAU patients collaborated. There was no evidence of selective drop-out. A complete case, repeated measure analysis of variance on the basis of intention-to-treat showed that, 5 years post-treatment, MBTp patients still scored better on social functioning compared to baseline [η_p_^2^ = .25, *p* = .01], whereas TAU patients did not [η_p_^2^ = .01, *p* = .67], with a significant difference between the conditions [η_p_^2^ = .10, p = .03]. A sensitivity analysis with linear mixed models, however, showed weaker evidence for an additive effect of MBTp over TAU on social functioning 5 years post-treatment, *F* = 3.731, *p* = .06. MBT patients also showed a greater improvement in one aspect of mentalizing, understanding of social causality [η_p_^2^ = 0.17, *p* = .04], but not other aspects of mentalizing.

**Conclusion:**

The results suggest a durable effect of MBTp.

## Introduction

Non-affective psychotic disorder (NAPD) is an umbrella term comprising psychotic disorders, such as schizophrenia, delusional disorder, schizoaffective disorder, and psychotic disorder not otherwise specified, which are characterized by positive symptoms – hallucinations and delusions – and significant impairments in social functioning. While positive symptoms can often be managed effectively with antipsychotic medication, research suggests that social dysfunction is not (Bellack et al., 2004) or is only partially responsive to pharmacological treatment (Devoe, Farris, Townes, & Addington, 2019; Raghavan, Ramamurthy, & Rangaswamy, 2017). Moreover, research suggests that functional impairment tends to be unaffected by symptomatic improvement through pharmacological therapy (Hamm et al., 2018). Additionally, social cognition – the process of how people perceive, interpret, and respond to social information that underlies social functioning – seems either unaffected or may even be hampered by some types of medication (Haime et al., 2021). This disparity frequently leads to a significant gap between symptom severity and functional impairment, sometimes referred to as the ‘symptom-disability gap’ (e.g. Birchwood, 2009). Nevertheless, a review of several studies concluded that functional recovery is achievable, with the recovery rate for adequate social functioning ranging from 21% to 53% (Leonhardt, Horan, & Lysaker, 2017).

In addition to pharmacological influences, however, social factors – such as a sense of connectedness (Eisenstadt, Monteiro, Diniz, & Chaves, 2012; Hendryx, Green, & Perrin, 2009) and the presence of social support (Norman, Windell, & Manchanda, 2013; Thomas, Muralidharan, Medoff, & Drapalski, 2016) – appear to play a pivotal role in both subjective and objective functional recovery. Consequently, developing treatments to improve social functioning in NAPD has become a key objective in psychosis research (e.g. Holthausen et al., 2007).

An important challenge for nonpharmacological treatments is the generally impaired level of mentalizing in this patient group (Brent & Fonagy, 2014; Weijers et al., 2024), as many forms of psychotherapy tend to rely on a patient’s ability to reflect on their own states and to understand and, to a degree, trust in the therapist’s intentions. Mentalizing, a fundamental component of social cognition, is defined as the process through which we infer one’s own and others’ mental states (Bateman & Fonagy, 2004). Impairments in various aspects of mentalizing have been identified, including the ability to infer others’ mental states (theory of mind) and to describe one’s own and others’ emotional states (see Sprong, Schothorst, Vos, Hox, & van Engeland, 2007; Kohler et al., 2010; O’Driscoll, Laing, & Mason, 2014 for overviews). Such deficits contribute negatively to functional recovery (Bechi et al., 2019; Fett et al., 2011). Mentalizing impairments have also been evidenced in relation to premorbid risk (Debbané et al., 2016), have been identified in ultra-high-risk states and are associated with higher levels of conversion to first-episode psychosis (Boldrini et al., 2019, 2020). Finally, recent research indicates that mentalizing may in fact deteriorate in some patients over the course of the disease if left untreated (Weijers et al., 2024).

Fonagy and Bateman (2006) have argued that sustaining mentalizing capacity may be critical in managing severe mental health conditions, as it aids emotional regulation and social interaction. Mentalization-based treatment (MBT; Bateman & Fonagy, 1999) was developed to enhance mentalizing and has become an established, evidence-based intervention for borderline personality disorder (BPD). MBT aims to orient attention toward emotions and to help identify, label, and reflect on those emotions. In this way MBT is thought to help individuals manage emotions more effectively, in turn yielding an improved ability for social cognitive perspective taking. Through this increased ability for perspective taking, mentalizing is thought to enhance social learning, which, when generalized beyond therapy groups, is thought to critically contribute to deeper, more trusting relationships and thus aid social functioning (Fonagy & Allison, 2014).

Indeed, research with regard to BPD has demonstrated that MBT can lead to durably reduced symptoms and improved social functioning up to 5 years following the conclusion of the treatment (Bateman, Constantinou, Fonagy, & Holzer, 2021). Subsequent research also observed that MBT can enhance mentalizing in self-harming adolescents (Rossouw & Fonagy, 2012) patients with borderline personality disorder (De Meulemeester, Vansteelandt, Luyten, & Lowyck, 2018), psychotic disorders (Weijers, Ten Kate, Viechtbauer et al., 2021), and a variety of personality disorders (Rizzi, Weijers, Kate, & Selten, 2024).

Given the observed mentalizing impairments in NAPD (e.g. Sprong et al., 2007), which in turn have been linked to social dysfunction in this patient group (Bechi et al., 2019; Fett et al., 2011), MBT has been recognized as a potentially promising treatment. Preliminary studies and theoretical papers proposed its potential benefits along the continuum of psychosis (Brent & Fonagy, 2014; Bröcker, von Haebler, Lempa, & Montag, 2023; Dangerfield & Brotnow Decker, 2023; Debbané et al., 2016; Lana et al., 2020; Weijers et al., 2016). Weijers and colleagues (2020) conducted a randomized controlled trial to examine the effectiveness of MBT for psychotic disorder (MBTp) and observed a significant and sustained increase in social functioning compared to treatment as usual (TAU) 6 months after treatment termination. Additionally, participants in this group performed better on several indices of mentalizing capacity compared to TAU. While these initial results showed promise, it remained unclear whether MBTp yields sustainable results over the years. This question is especially pertinent, given the long-term effects observed in MBT for BPD (Bateman et al., 2021). In line with Bateman et al. (2021), we conducted a 5-year follow-up study to evaluate the long-term effects of MBTp on social functioning and mentalizing capacity in NAPD. We hypothesized that MBTp participants show sustained improvements in social functioning and mentalizing capacity 5 years post-treatment.

## Methods

### Study protocol

The present study is a follow-up study from an RCT (Weijers, Ten Kate, Viechtbauer, et al., 2021). The original study received approval from the Medical Ethics Committee of Maastricht University (no. 1303066). The current study – conducted 5 years after treatment termination – was again registered in advance at and approved by the Medical Ethics Committee of Maastricht University (no. 20202267). A total of four measurements were taken from the start of the original study up to the current study: at baseline, directly post-treatment, 6 months post-treatment and 5 years post-treatment.

### Participants

As described before (Weijers et al., 2016), inclusion criteria required participants to have been undergoing treatment for non-affective psychotic disorder (NAPD) for at least 6 months, but no more than 10 years, and to be between 18 and 55 years of age. Exclusion criteria included borderline intellectual functioning (as diagnosed by a psychiatrist or psychologist), illiteracy, insufficient proficiency in the Dutch language, and addiction to drugs or alcohol requiring inpatient detoxification. Please refer to table 1 for participant demographics.

**Table 1.** Demographics and clinical characteristics of patients at 5-year follow-up

Variable	TAU, mean (s.d.)	*N*	MBTp, mean (s.d.)	*N*	*p* ^a^
Age in years	37.93 (8.78)	23	37.80 (8.38)	23	0.96
Age of onset	26.71 (9.11)	23	25.76 (7.51)	23	0.60
Duration of illness	12.33 (3.34)	23	11.89 (3.35)	23	0.66
Gender					0.13
Male, #		17		11	
Female, #		6		12	
Diagnosis (DSM-IV-TR)					0.32
Schizophrenia		14		13	
Schizoaffective disorder		3		5	
Psychotic disorder N.O.S.		4		4	
Brief psychotic disorder		0		1	
Delusional disorder		2		0	

a Based on independent samples *t* tests for continuous variables and χ^2^ tests for categorical ones.

### Treatment

*Treatment as usual: functional assertive community care.* For a more elaborate description of the FACT approach, please refer to Supplementary Appendix I.

*Experimental treatment:* For a full description of MBTp, please refer to our previous publications (Weijers et al., 2016; Weijers, Ten Kate, Debbané, et al., 2020; Weijers, ten Kate, Siecker, & Noij, 2023) and Supplementary Appendix I.

*Equivalence of treatment conditions:* As described in the original trial paper (Weijers, Ten Kate, Viechtbauer, et al., 2021), the two treatment conditions were comparable but not completely equivalent with regard to dosage and type of therapy. Participants in the MBTp group received both individual and group therapy, whereas those in the FACT group received more cognitive behavioral therapy for psychosis and individual placement and support between the baseline and post-treatment assessments.

### Blinding procedure

All measurements were conducted by researchers that were blind to treatment allocation. Blinding was only broken once, with the measurement taken by another assessor blind to treatment allocation.

### Measurement instruments

*Primary outcome:* Social functioning was measured using the Social Functioning Scale (SFS; Birchwood et al., 1990). This questionnaire assesses seven dimensions of social functioning: social withdrawal, interpersonal communication, independence (competence), independence (performance), recreational activities, social activities, and employment Subscale scores were averaged to create an overall social functioning score, with higher scores indicating better social functioning (range: 59.7–134.9). The SFS is noted for its reliability, responsiveness to change, and construct validity (Birchwood et al., 1990).

*Secondary outcomes:* Two measures were used to measure several aspects of mentalizing capacity. Firstly, narratives gathered with the Thematic Apperception Test (TAT; Murray, 1943) were scored with the Social Cognition and Object Relations System (SCORS; Westen, 1991). The SCORS (Westen, 1991) is a clinician-rated system designed to assess the quality of an individual’s internal representations of self and others, as expressed in narrative material such as Thematic Apperception Test narratives. The SCORS comprises four interrelated dimensions that together reflect the maturity and integration of object relations and social cognition. These dimensions include (i) the complexity of representations of people, (ii) the affective tone of relationships, (iii) the capacity for emotional investment, and (iv) the understanding of social causality. Each scale is rated on a five-point continuum, with lower scores reflecting fragmented, one-dimensional, or malevolent representations and higher scores indicating differentiated, reciprocal, and emotionally integrated depictions of self and others. Six pictures from the Thematic Apperception Test (TAT) were administered, and participants’ responses were recorded and transcribed verbatim for analysis. When coded using the SCORS (Westen, 1991), TAT narratives have demonstrated strong reliability and validity as measures of social cognition and object relations (Hibbard, Mitchell, & Porcerelli, 2001; Meyer, 2004). According to Luyten, Fonagy, Lowyck, and Vermote (2012), the TAT remains one of the few assessment tools that capture nearly all aspects of mentalization, encompassing both affective and cognitive components.

We included three dimensions of mentalizing capacity using the SCORS: complexity of representations; understanding of social causality and capacity for emotional investment. We excluded the affective tone of relationships dimension because previous studies have shown it to be less internally consistent (Hibbard et al., 2001) and less responsive to treatment (Rizzi et al., 2024; Weijers, Ten Kate, Viechtbauer, et al., 2021). Complexity of representations reflects an individual’s ability to differentiate between self and other perspectives and to construct coherent, psychologically rich portrayals of people that integrate motives, emotions, and behaviors over time. Understanding of social causality measures the extent to which interpersonal behavior is explained through psychologically meaningful cause-and-effect reasoning. It evaluates whether the respondent can attribute actions to underlying mental states – such as intentions, desires, or emotions – rather than to purely external or arbitrary factors. Finally, the Capacity for Emotional Investment scale assesses the quality, depth, and maturity of a person’s emotional ties to others, self, and ideals. It reflects how an individual experiences, values, and sustains emotional connections – whether those connections are mutual, empathic, and enduring, or superficial, self-serving, and transient.

All SCORS interviews were conducted by master’s students in Clinical Psychology who had received a 2-day training by co-author EEB. Interrater reliability was assessed by means of recorded narratives and rated independently by all raters. Inter-rater reliability was acceptable for complexity of representations (Cohen’s κ = 0.7), good for understanding of social causality (Cohen’s κ =0.8), and excellent for capacity for emotional investment (Cohen’s κ =0.9).

Secondly, the Hinting Task (Corcoran, Mercer, & Frith, 1995) was used to assess theory of mind, that is, the ability to understand and infer the intentions, beliefs, or desires of others. The task specifically measures the ability to comprehend indirect hints in social interactions. The task involves a series of short scenarios in which a character drops a hint about their true intentions, which are to be inferred by the participants. Scores range from 0 and 20, with higher scores indicating greater theory of mind. The Hinting Task is a widely used task to measure Theory of Mind in the context of NAPD research, has been extensively evaluated with respect to its test–retest reliability, suitability as a repeated measure, relation to functional outcomes, and internal consistency and was identified as one of the few instruments demonstrating robust psychometric properties across all evaluation criteria (Pinkham, Penn, Green, & Harvey, 2015).

### Statistical analyses

*Main analysis.* As described in the original study protocol, to estimate the effect of the treatments at each time point, we conducted a complete case, repeated-measures analyses of variance for the outcome variables, adhering to the intention-to-treat principle. These analyses included baseline scores (T0), post-treatment scores (T1), 6 months post-treatment scores (T2) and 5 years post-treatment scores (T3) as dependent variables, and treatment condition (either TAU or MBTp) as independent variable. These analyses were conducted for social functioning and the secondary mentalizing outcome variables: understanding of social causality, complexity of representations, capacity for emotional investment and theory of mind. We also examined the specific effect of treatment condition between just the baseline (T0) and 5-year follow-up scores (T3).

*Handling of missing data.* Long-term follow-up studies are often plagued by missing data and selective participant attrition (Sullivan et al., 2017). Attrition may be influenced by factors like level of functioning before the start of the treatment and the differing conditions of treatment. Initially, our preregistered protocol suggested multiple imputation (MI) as a strategy for handling participant attrition. However, we decided to deviate from this plan because MI becomes unreliable when the proportion of participant attrition exceeds approximately 40% (Jakobsen, Gluud, Wetterslev, & Winkel, 2017). With 45% of missingness, linear mixed models (LMMs) were preferred over MI in order to make more robust use of the available data while minimizing the risk of biased parameter estimates due to excessive imputation. Therefore, in addition to a complete case analysis of the original data, LMMs were chosen as secondary sensitivity analyses because they are well suited for repeated-measures data with incomplete observations. Unlike traditional repeated-measures ANOVA, LMMs can accommodate unbalanced datasets and handle missing outcome data under the assumption that data are missing at random (MAR), without requiring listwise deletion or imputation of missing values (Gueorguieva & Krystal, 2004; Twisk, 2013).

## Results

### Participant flow and baseline characteristics

The study initially included 84 patients with NAPD, randomly assigned to MBTp or TAU. The allocation resulted in 42 patients per group. Five years after the end of treatment 46 patients, or 55% of the total number of starters, participated in the follow-up study. Attrition was equivalent in both treatment conditions (*N* = 23 in each condition, respectively). There were no significant differences in social functioning at baseline between the two groups (*p* = .40). Additionally, there were no significant differences in social functioning between those who dropped out of the study and those who did not, at either baseline, post-treatment, or at the 6-month follow-up (all *p*s > .38). Furthermore, neither within the MBTp condition (all *p’*s > .67) nor in the TAU condition (all *p*’s > .11) were there significant differences in social functioning between those who later dropped out and those who did not at either baseline, post-treatment, or the 6-month follow-up mark. In short, no evidence of selective drop-out was observed, at least with regards to social functioning.

*Statistics information:* The analyses mentioned below were conducted using SPSS statistics version 25 by IBM. Means and standard deviations of all tests can be found in Supplementary Appendix I. Assumptions of all analyses were tested and are detailed in Supplementary Appendix II.

*Complete case analysis of primary outcome*:* Social Functioning.* We conducted a complete case, repeated measures analysis on the basis of intention to treat, with time and treatment condition (MBTp or TAU) and their interaction effect as independent variables and social functioning as dependent variables. We considered the overall effects including all measurement points (T0, T1, T2, and T3) and the specific difference between baseline (T0) and 5-year follow-up (T3).

**Figure 1.**
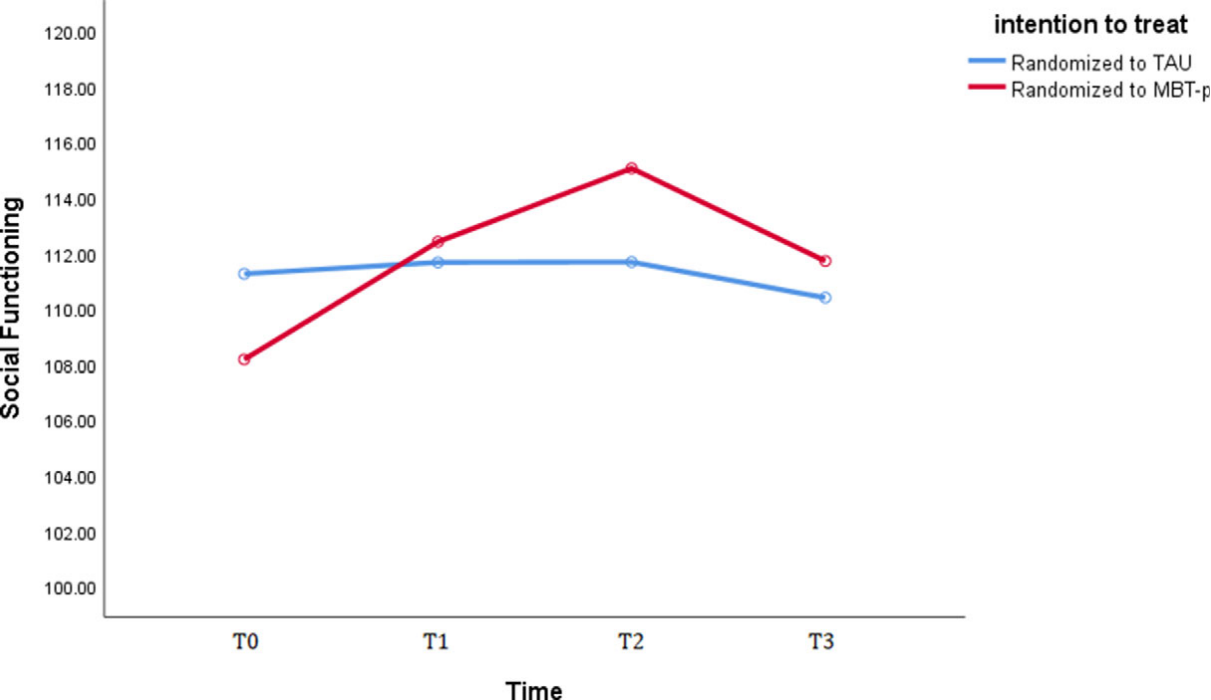
Performance on the Social Functioning Scale as a function of time and treatment condition. *Note:* T0 = baseline; T1 is post-treatment; T2 is 6-month follow-up; T3 is 5-year follow-up. TAU, treatment as usual (functional assertive community treatment); MBTp, mentalization-based treatment for psychotic disorders.

*Overall improvement over time:* Results of the repeated-measures analysis with all measurement points combined revealed that patients in the MBTp condition showed a significant and large gain in social functioning from baseline through post-treatment and 6-month follow-up and up to the 5-year follow-up mark.[*F*(1,20) = 4.92, η_p_^2^ = .45, *p* = .01] whereas patients in the TAU FACT condition did not, [*F*(1,20) = 0.66, η_p_^2^ = .10, *p* = .59]. There also was a significant time x treatment condition effect on social functioning [F(1,20) = 6.44, η_p_^2^ = .19, p = .04], meaning that patients in the MBTp condition showed a significantly greater gain in social functioning when taking all measurement points into account in comparison to the TAU condition. The benchmarks established by Cohen (1988); 0.01 = small; 0.06 = moderate, 0.14 = large) mean that this effect can be considered large. Please refer to figure 1 below for a visual representation of treatment effects over time.

*Durability of improvement:* Regarding the specific difference in social functioning gains between just baseline (T0) and 5-year follow-up (T3), results revealed that patients in the MBTp condition showed a significant and large gain in social functioning from baseline up to 5 years post-treatment [*F*(1,20) = 7.24, η_p_^2^ = .25, *p* = .01] compared to patients in the TAU condition who did not [*F*(1,20) = 0.19, η_p_^2^ = .01, *p* = .67]. There was a significant time x treatment condition effect [F(1,20) = 5.08, η_p_^2^ = .10, p = .03], meaning that patients in the MBTp showed a moderately large and significantly greater gain in social functioning 5 years after treatment termination when compared to those in the TAU condition.

### Complete case analysis of s*econdary outcomes: aspects of mentalizing capacity*

*Overall improvement over time:* Results of the repeated-measures analyses with understanding of social causality, complexity of representations, capacity for emotional investment, and theory of mind as dependent variables and with all measurement points taken into account, revealed that patients in the MBTp condition showed a large and significantly greater increase in understanding of social causality compared to the TAU condition [*F*(5,30) = 3.22, η_p_^2^ = 0.17, *p* = .04]. Whereas patients in the MBTp condition showed a large increase in understanding of social causality [*F*(1,16) = 3.63, η_p_^2^ = 0.17, *p* = 0.04], patients in the TAU condition did not, [*F*(1,14) = 0.81, *p* = 0.5]. No significant time x treatment condition interaction effects were observed on either complexity of representations, capacity for emotional investment or theory of mind [all *p*’s > .39]. As described in Supplementary Appendix II, not all statistical assumptions were met in the case of theory of mind (hinting task). A secondary LMM analysis was therefore conducted, which also showed a non-significant time x treatment condition interaction effect (*p* = .89).

*Durability of improvement:* A comparison of the differences between just baseline (T0) and at 5-year follow-up (T3) revealed no significant differences in any of the aspects of mentalizing capacity. There was also no overall significant treatment condition x time interaction effect on either understanding of social causality, capacity for emotional investment, complexity of representations, or theory of mind (all p’s > .09).

### Sensitivity analysis of the primary outcome with linear mixed models

A secondary linear mixed model with repeated observations at either two (T0, T3) or four time points (T0, T1, T2, and T3) was conducted as a sensitivity analysis to account for the amount of attrition. Because repeated observations within a participant are correlated, the assumption of independent residuals required for standard regression is violated; therefore, a multilevel modeling approach was applied, with multiple observations (level 1) nested within subjects (level 2; Hox, 2010). Mixed-effects linear regression models were used to test the hypothesis that social functioning would increase more in the MBTp condition than in the TAU condition. Random intercepts and random slopes for social functioning were specified at the participant level, with an unstructured variance–covariance matrix for the random effects. *Overall improvement over time:* When including all timepoints (T0, T1, T2, and T3), the effect of time on social functioning was statistically significant for participants in the MBTp condition *F*(3, 97.92) = 13.33, *p* < .01, indicating that social functioning improved significantly over the whole study period. In the TAU condition, the effect of time was not significant, *F*(3, 53.48) = 0.57, *p* = .45, indicating little change in social functioning across the four assessment points. Finally, the interaction effect of time and treatment condition was significant in the four-time-point model, *F*(3, 191.73) = 4.91, *p* = .028, showing that the change in social functioning was markedly larger in the MBTp condition.

Durability of improvement: Regarding the specific difference in social functioning gains between just baseline (T0) and 5-year follow-up (T3), the effect of time on social functioning was not significant, *F*(1, 28.95) = 0.17, *p* = .90, for patients in the TAU-group, indicating that social functioning did not change significantly over the study period. For participants randomized to MBTp, the effect of time was significant, *F(*1, 30.77) = 7.81, *p* = .01, suggesting a durably improved social functioning at the 5-year mark. However, the interaction effect of time and treatment condition failed to reach statistical significance in the two-time-point model, *F*(3, 59.96) = 3.731, *p* = .06, suggesting weaker support for the hypothesis that the durability of improvement in the MBTp group is greater than that of the TAU group.

## Discussion

### Summary of results

This study examined the effects of MBTp 5 years after termination. A complete case, intention-to-treat repeated measures analysis showed that patients in the MBTp condition showed a greater increase in social functioning. The gains in social functioning proved to be robust: patients in the MBTp condition still showed a large increase from baseline up to 5 years after treatment, whereas the patients in the TAU condition did not. The difference was moderately large and significantly greater for the MBTp group compared to TAU. The results also seem to be clinically relevant as well; patients in the MBTp group showed great improvements from baseline (*M* = 108.94) up to 6 months after treatment (*M* = 115.402 and to a lesser extent up to 5 years after treatment (*M* = 111.87) with a level of functioning in between those of schizophrenia patients (*M* = 102.71) and non-patient controls (M = 119.64), as observed by Birchwood et al. (1990).

Additionally, a number of sensitivity analyses with linear mixed models also revealed an overall improvement over time and a condition interaction effect, indicating that patients in the MBTp condition showed greater overall improvement in social functioning compared to those receiving TAU. Patients in the MBTp group also showed a sustained improvement in social functioning at the 5-year post-treatment mark, whereas TAU patients did not. However, evidence for a significant difference in durability of treatment effects between these conditions was weaker.

The sensitivity analyses largely showed similar results to the complete case analyses, supporting the robustness of findings. However, the differences regarding the durability of the treatment effects introduce more uncertainty and show that the primary analysis might suffer from participant attrition. Having said that, both the primary and secondary analyses point in the same direction. Also, the lack of a difference in social functioning at any measurement point between patients who dropped out and those who did not, and the identical number of participants who dropped out in each treatment condition (*N* = 19), do not corroborate the notion of selective attrition. Finally, participants showed greater overall improvement on the understanding of social causality dimension of the SCORS (Westen, 1991) but not on other measures of mentalizing. The improvement in understanding did not prove to be durable however. This finding is theoretically consistent with the central mechanisms of MBT, which aim to enhance the ability to infer and reflect on psychological motives, intentions, and emotions underlying the social behavior (Fonagy & Bateman, 2006). MBTp directly targets understanding of social causality by fostering curiosity, perspective taking, and tolerance for ambiguity, thereby recalibrating the biased or hyper- or hypo-mentalized attributions common in psychosis (Frith, 1992). Through repeated exploration of misunderstandings and ruptures within a safe therapeutic relationship, patients practice drawing psychologically plausible connections between internal affective states and observable behavior. In contrast, other SCORS dimensions – complexity of representations and capacity for emotional investment – and the Hinting Task dimension of theory of mind (Corcoran et al., 1995) likely index more stable, trait-like structures that evolve gradually through long-term relational experience (Westen, 1991). Thus, while MBTp appears particularly effective in enhancing flexible, moment-to-moment social-causal reasoning, deeper structural aspects of object representations, and abstract mental-state reasoning may require longer-term treatment beyond the time frame of the intervention.

*Comparison with other studies.* In their review, Green, Horan, and Lee (2015) noted that 100 years of research and therapy had not led to an increase in social functioning of NAPD patients. The results of the current study certainly do not paint so bleak a picture, as they suggest that social dysfunction in NAPD can in fact be treated effectively and potentially even durably, like in other studies examining MBT for non-psychotic disorders (Bateman et al., 2021; Bateman & Fonagy, 1999, 2001).

These findings are consistent with growing evidence underscoring the clinical value of social-cognitive approaches in the treatment of psychosis. A meta-analysis by Turner, van der Gaag, Karyotaki, and Cuijpers (2017) demonstrated that social-cognitive interventions targeting negative symptoms and social dysfunction in psychosis outperform TAU. More specifically, in relation to MBTp, Dangerfield and Brotnow Decker (2023) found that an integrated AMBIT–MBT approach for engaging young people with psychotic presentations led to significant improvements in adaptive functioning (in the form of reduced school absenteeism). The results of the current study suggest that social functioning can be improved, possibly even up to the 5-year post-treatment mark. However, the effects of treatment on social functioning and understanding of social causality did diminish after 5 years. These results deviate from those observed for BPD, where the effect had not only remained but had even increased after 5 years (Bateman et al., 2021; Bateman & Fonagy, 1999, 2001). Below we offer some suggestions as to why initial gains from MBTp were at best maintained in the NAPD group.

*Relatively short duration of treatment.* Given the potentially progressive nature of NAPD and more severe mentalizing deficits in NAPD patients compared to BPD patients (Ridenour et al., 2019), MBTp may require more time than MBT for BPD. Patients may also require prolonged therapy to allow them enough time to feel secure enough while exploring their emotional states and to maintain achieved gains. Moreover, aftercare in the form of group therapy was part of the original MBT program for BPD (Bateman & Fonagy, 1999). It can be considered an omission in the current study that patients did not receive such aftercare.

*Lack of conceptual adaptations in working with NAPD patients.* At the start of the trial in 2013, there was a scarcity of conceptual work describing approaches to mentalization-based work with patients with NAPD. Since then, several papers have considered the theoretical background of MBT (Bateman et al., 2023; Debbané & Bateman, 2019; Salaminios, Barrantes-Vidal, Luyten, & Debbané, 2024). and have proposed technical adaptations. For example, Bröcker and Montag (2023) have proposed technical adaptations with a particular focus on implicit ways to regulate closeness and distance in the relationship between the patient and therapist to make treatment more tolerable to the patient, at least in the initial phase.

*Natural decline in mentalizing in NAPD.* Finally, one reason why growth was stunted at follow-up may be the often-progressive nature of NAPD and, in particular, a potential for a decline in mentalizing over time. In a recent study we observed that several aspects of mentalizing tended to decline in patients with schizophrenia if left untreated (Weijers et al., 2024). This does pose the question whether – like pharmacological treatment – therapy should be offered on a maintenance basis in NAPD.

Declines in mentalizing are likely influenced by multiple factors, but social isolation is thought to play a major role. Psychosis can severely disrupt social relationships through hospitalizations, negative and positive symptoms, stigma, and medication side-effects (Pereira & Debbané, 2018), leaving patients with reduced social networks due to lost friendships, romantic relationships, or employment. Mentalization theory emphasizes that progress depends less on therapy sessions themselves than on what occurs between them. By enhancing mentalizing and restoring epistemic trust, patients may re-engage in meaningful communication, better evaluate their circumstances, and revise cognitive models based on social feedback (Fonagy & Allison, 2014). The reduction of social networks likely reduces opportunities for reciprocal interpersonal feedback (Pereira & Debbané, 2018); may limit opportunities to challenge entrenched beliefs (Lincoln et al., 2014) and thus contributes to poorer treatment outcomes (Lieberman, Dixon, & Goldman, 2013; Lincoln et al., 2014). Importantly, quality and warmth of bonds – rather than number of contacts – seems to be key here, as it protects against relapse (Degnan et al., 2018; Lee, Barrowclough, & Lobban, 2014; Vogel et al., 2021) and aids recovery (van Bussel et al., 2021).

Finally, neurocognitive decline may also have stymied (durable) therapeutic effects on social functioning and mentalizing capacity. A recent study observed that neurocognitive decline in SCZ averaged 16 IQ points from adolescence onward and progressed more rapidly after psychosis onset (Jonas et al., 2022). This neurocognitive decline is potentially impactful for mentalizing, as neurocognitive aspects such as attention, working memory, and executive functioning are also crucial in mentalizing. This seemingly progressive decline in neurocognition may have stymied gains in social functioning, as it has been linked to social isolation in NAPD (Badcock et al., 2015; Yu et al., 2024).

### Strengths and limitations

The present study employed a rigorous research design, incorporating randomization, consideration of baseline performance, and the use of blinded raters. Furthermore, it is the first study to investigate the long-term efficacy of MBT in NAPD and one of the few studies to assess whether MBT enhances mentalizing capacity (see Rizzi et al., 2024, for another example). Additionally, intensive supervision was provided to ensure that practitioners adhered to the MBT model. Furthermore, the patient groups were well-matched on variables such as age, educational level, social functioning, aspects of mentalizing capacity, symptom severity, and medication use at baseline (see Weijers, Ten Kate, Viechtbauer, et al., 2021). Patients in the MBTp condition only scored significantly higher on depression and anxiety at baseline (Weijers, Ten Kate, Viechtbauer, et al., 2021). Additionally, a notable strength of the present study is the use of task-based measures of mentalizing rather than relying solely on self-report questionnaires, thereby minimizing the effects of social desirability, self-presentation, and introspective bias that often confound questionnaire data (Luyten et al., 2012). Finally, the TAU condition, which includes psychiatric counseling, social work, individual placement, and support training and also could include CBTp and/or EMDR can be considered a highly active control condition. That significant differences were found on some outcome variables may be considered all the more striking.

However, several limitations must also be acknowledged. First, a substantial drawback of the current study is the relatively large amount of participant attrition at 5-year follow-up (45%). Although this rate of attrition is in line with other NAPD studies – with attrition rates ranging from 36% to 90% (see Homman, Smart, O’Neill, & MacCabe, 2021 for an overview) – it *does* pose statistical limitations on generalizability. We attempted to overcome this limitation by complementing the complete case analysis with linear mixed models, potentially adding to the robustness of our conclusions. The absence of any evidence for selective drop-out also supports the validity of the results. Still, given the participant attrition, the complete case analysis results should be interpreted with caution as well and perhaps even be treated as hypothesis generative rather than confirmative results (Jakobsen et al., 2017). Third, as described before, the two treatment conditions were not equal intervention-wise, complicating the determination of whether the observed differences were genuinely a result of the interventions themselves. However, given that the TAU condition included substantially more cognitive behavioral therapy and individual placement and support than MBTp, whereas the MBTp condition included individual and group MBTp sessions, we believe that the treatment condition can be considered roughly equivalent. Fourth, the study predominantly assessed other-oriented, cognitive forms of mentalizing, while it has been argued that patients with NAPD are most severely impaired in self-oriented, affective mentalizing (Debbané et al., 2016). Fourth, epistemic trust – that is, a person’s willingness to consider new knowledge as trustworthy and relevant – was not taken into account in the current study. Epistemic trust has been theorized to underlie social learning and to potentially be a major driver of MBT treatment effect (Fonagy & Allison, 2014), perhaps more so than an increase in mentalizing capacity. It should be included in future research. Fifth, the Hinting Task has been criticized for a ceiling effect, particularly among high-functioning patients (e.g. Roberts & Penn, 2009), necessitating a cautious interpretation of the related findings.

## Conclusion

The results of the current 5-year follow-up study suggest that MBTp has a greater overall effect on social functioning and at least one type mentalizing than TAU. Results from the primary analysis also suggest that gains in social functioning are durable in the MBTp-condition up to the 5-year post-treatment mark. However, some uncertainty remains about the potential of selective attrition influencing these results, as shown by the sensitivity analysis. Still, based on our previous (Weijers, Ten Kate, Viechtbauer, et al., 2021) and current studies, and the work of our colleagues (Leonhardt et al., 2017; Thomas et al., 2016), we feel confident that our results show the value of targeting social recovery in the non-pharmacological treatment of NAPD. It is time to abandon doom and gloom and examine and implement ways, like MBTp, to durably aid social recovery in NAPD.

## Supporting information

10.1017/S0033291725102821.sm001 Weijers et al. supplementary material Weijers et al. supplementary material

## Supplementary material

The supplementary material for this article can be found at http://doi.org/10.1017/S0033291725102821.
