# Supplementary material for: Long-term effects of mentalization-based treatment for psychotic disorder: a 5-year follow-up of a multi-center, randomized-controlled trial
Source: Psychol Med. 2026 Jan 9;56:e12. doi: 10.1017/S0033291725102821 (PMC12885340; doi:10.1017/S0033291725102821)
Supplement: Weijers et al. supplementary material [file S0033291725102821sup001.docx]

**Supplementary Material**

***Appendix I: Provided treatments***

*Flexible Assertive Community Treatment:*

Participants received care based on the Dutch multidisciplinary guideline for schizophrenia (van Alphen et al., 2012) and the Functional Assertive Community Treatment (FACT) model. The aim of Functional Assertive Community Treatment (FACT) is to reduce hospitalizations and to improve quality of life by providing a multidisciplinary team that delivers personalized care in the community, focusing on areas like housing, employment, social skills, and health management.

The FACT model was developed in the Netherlands in the early 2000s designed to address the full continuum of care for individuals with severe mental illness (SMI), including NAPD (van Veldhuizen, 2007; van Veldhuizen & Bähler, 2013). FACT generally employs a broad range of disorders, encompassing all people with SMI within a defined catchment area. The model was conceived to provide both continuity and flexibility of care, ensuring that clients do not have to move between teams as their level of need fluctuates.

The aim of FACT is to reduce hospitalizations and to improve quality of life by providing a multidisciplinary team that delivers personalized care in the community, focusing on areas like housing, employment, social skills, and health management. FACT typically emphasizes more behavioral, supportive, and symptom- or crisis-management interventions, aiming to offer solutions to different problems.

At the heart of FACT lies a multidisciplinary community-based team—typically comprising psychiatrists, psychologists, psychiatric nurses, social workers, peer specialists, and employment or housing specialists—responsible for approximately 200 clients per team within a geographic area of roughly 40,000–50,000 inhabitants (van Veldhuizen & Bähler, 2013). The model’s flexibility comes from its ability to “up-scale” and “down-scale” the intensity of support within the same team. When a client is relatively stable, care is provided through individual case management and recovery-oriented interventions. When a client’s condition deteriorates, the team collectively shifts to a shared-caseload, outreach-based approach, providing daily, assertive contact and crisis management until stability is restored. This fluid adjustment aims to prevent fragmentation and to promote trust through consistent therapeutic relationships.

A distinctive feature of FACT is its daily multidisciplinary “FACT board” meeting, where the team reviews clients currently requiring high-intensity intervention. This meeting enables rapid response to emerging crises and ensures that knowledge about each client is shared among all team members. FACT integrates treatment, rehabilitation, and recovery-supporting activities into one service structure, aiming to promote both clinical and social recovery (van Veldhuizen & Bähler, 2013). Services extend beyond symptom management to include employment and housing support, social skills training, and engagement with family or informal networks.

Within FACT, treatment for psychosis follows the Dutch multidisciplinary guideline for schizophrenia and related disorders, which integrates pharmacological, psychological, and psychosocial interventions (van der Lee et al., 2016). Medication remains the foundation of acute symptom control, with antipsychotic drugs prescribed to reduce positive symptoms and prevent relapse. Pharmacological treatment is complemented by psychoeducation, emphasizing adherence, side-effect management, and recognition of early warning signs. In line with recovery-oriented practice, psychological therapies are increasingly central to FACT care. Cognitive Behavioural Therapy for psychosis (CBTp) is widely implemented and for clients with trauma histories, trauma-focused therapies such as EMDR or imaginary exposure are used (van den Berg et al., 2015). Similarly, social recovery and rehabilitation interventions—including Individual Placement and Support (IPS) for competitive employment—are integral components of FACT and mandated in its fidelity standards (van Veldhuizen & Bähler, 2013). These interventions aim to restore social roles and enhance quality of life beyond symptom remission.

Finally, Dutch psychosis care under FACT emphasizes physical health monitoring and relapse prevention planning. People with psychosis face elevated risks of cardiovascular disease and metabolic syndrome; therefore, regular screening and lifestyle interventions are built into routine care. Crisis and relapse plans are co-produced with clients and families to ensure rapid access to support when early warning signs emerge.

*Mentalization-Based Treatment for Psychosis*:

In the experimental condition, next to treatment according to the FACT-model, participants received Mentalization-Based Treatment for Psychotic Disorder (MBTp). The MBTp intervention involved biweekly individual and weekly group psychotherapy sessions for a duration of 18 months, focusing on enhancing mentalizing capacity, particularly under stress, in order to reduce psychopathology and improve social functioning (Fonagy & Bateman, 2006). Adherence was monitored through weekly supervision sessions, and an adherence check used Karterud’s (2015) MBT Adherence Scale: three videotaped sessions were independently rated by an MBT supervisor/trainer and judged as adequate to good in fidelity to MBT principles (Weijers et al., 2020).

Mentalization-Based Treatment for psychosis (MBTp) represents a reworking of traditional MBT to accommodate to specific vulnerabilities of psychosis. While MBT was originally developed for borderline personality disorder (BPD) and aims to strengthen the capacity to understand oneself and others in terms of mental states (Bateman & Fonagy, 1999, 2006), MBTp emerged from the recognition that individuals with a psychosis vulnerability face unique challenges in emotional regulation, interpersonal trust, and reflective functioning (Weijers, ten Kate, Siecker, & Noij, 2020). Psychotic processes often involve distortions in self–other understanding (Debbané et al., 2016), which require a recalibration of the standard MBT model rather than an entirely new therapeutic approach.

At its core, MBTp retains MBT’s fundamental principles—an inquisitive, not-knowing stance, a focus on the here-and-now, attention to affect, and continuous calibration of intervention complexity to arousal—but it reorganizes these elements to meet the cognitive and emotional fragility characteristic of psychosis-proneness. The first major deviation lies in the sequencing and implicitness of therapeutic work. Whereas standard MBT often moves relatively quickly toward explicit reflection on the therapeutic relationship and may use mild confrontation or “challenges” to restore reflective functioning under stress, MBTp begins with a prolonged focus on implicit relational processes. Early sessions emphasize establishing joint attention, affect regulation, and a sense of safety, rather than direct attempts to enhance mentalizing. The therapist’s initial task is not to interpret or challenge but to create a trusting relational space. In some cases, this may include outreach beyond the therapy room—such as brief home visits or walks in the park—to maintain engagement and signal reliability.

This emphasis reflects the recognition that epistemic mistrust, or difficulty in seeing others as reliable sources of information, is particularly pronounced in psychosis due to early attachment disruptions and traumatic experiences (Gumley, Taylor, Schwannauer, & MacBeth, 2014; Varese et al., 2012). To counter this, MBTp therapists adopt a deliberately humble stance—“not-knowing,” validating, and authentically curious (Fonagy, Luyten, Allison, & Campbell, 2017)—seeking to become trusted collaborators in meaning-making rather than experts imposing interpretation. Perhaps the humble stance is even more important when working with psychosis-prone individuals, given their tendency to experience a sense of inferiority due to social isolation or perceived lower social status. Only when a foundation of safety and joint attention is in place does MBTp begin to scaffold explicit reflection.

In this second, more reflective phase, MBTp continues to diverge from MBT in its restraint around interpretive work of transference. Because affective overstimulation can rapidly erode reflective capacity, MBTp places stronger emphasis on arousal titration. MBTp therapists continuously adjust the intensity of interventions to keep clients within their “window of tolerance”– this is no way diverges from regular MBT (Fonagy & Bateman, 2006) – but it is thought that patients with NAPD are outside of the window of tolerance more often. Also note here that due to negative symptoms, such arousal may in fact be less evident in NAPD patients. The greater sensitivity to overstimulation also results in a different focus of interventions in practice. Whereas MBT for BPD uses exploration of the therapeutic relationship as an important arena for restoring mentalizing, MBTp introduces such exploration sparingly and only once stability is evident (Bateman, Fonagy, Campbell, Luyten, & Debbané, 2023). Instead of analyzing relational dynamics, MBTp prioritizes embodied mentalizing—helping clients notice, name, and regulate sensory-affective experiences before linking them to meaning. Therapists may offer tentative words for unarticulated feelings, functioning as linguistic “scaffolding” to reconnect bodily sensation with thought (Salaminios & Debbané, 2021). This approach acknowledges that many psychosis-prone clients lack access to emotional vocabulary and that premature cognitive exploration risks destabilizing fragile self-experience. MBTp therapists need to take more time to regulate arousal and validate emotions before trying to scaffold mentalizing (Bateman et al., 2023). Here, the clinical rhythm is slower and more circular: therapists repeatedly return to the anchor of the client’s immediate affective experience, fostering a greater affective stability that allows for gradual re-engagement with multiple viewpoints.

The interpersonal and social context of NAPD patients also tends to differ substantially from most BPD clients. Given that social exclusion and loneliness are major contributors to psychotic vulnerability (Selten, van der Ven, Rutten, & Cantor-Graae, 2013; Steenkamp, Weijers, Gerrmann, Eurelings-Bontekoe, & Selten, 2019), it must be noted that group therapy sometimes is the sole opportunity for patients to engage with others. This means that these sparse social interactions tend to hold greater significance for patients, yet this added meaning often intensifies feelings of pressure and anxiety. This places a greater emphasis on MBTp groups being safe, contained environments in which clients can tentatively re-engage with other minds. Therapists play a more active regulatory role—monitoring tension, avoiding expressed hostilities and sometimes explicitly “taking sides” to prevent social exclusion. Also, given a tendency for avoidant or disorganized attachment, temporary patient drop-out is more common. Keeping the patient in mind is crucial here, especially because he/she will at times not want to come into contact. Reminder calls or home visits after absences, is therefore more crucial here, reflecting the need to sustain fragile engagement.

Lastly, whereas regular MBT tends to favor an absence of psychopharmacological treatment where possible (Karterud, 2015), this is often more complicated for NAPD patients, as antipsychotics may prevent the rise of another psychotic episode. However, while antipsychotic medication may be necessary to reduce psychosis-proneness, excessive dosing can blunt affective access and impede mentalizing. Thus, MBTp rather opts to find an optimal balance of antipsychotics that on the one hand prevents psychotic break-throughs and on the other hand does not blunt affect too much, through careful coordination between client, MBT-therapists and prescriber.

In short, MBTp is not a different therapy so much as a reweighted application of MBT principles. It maintains the same therapeutic aims—fostering curiosity about one’s own and others’ minds—but with greater restraint, stronger grounding in bodily affect and slower pacing.

*References:*

Bateman, A., & Fonagy, P. (1999). Effectiveness of partial hospitalization in the treatment of borderline personality disorder: A randomized controlled trial. American Journal of Psychiatry, 156(10), 1563–1569.

Bateman, A., & Fonagy, P. (2006). Mentalization-based treatment: A practical guide. Oxford University Press.

Bateman, A., Fonagy, P., Campbell, C., Luyten, P., & Debbané, M. (2023). Mentalization-based treatment for personality disorders. Oxford University Press.

Castelein, S. (2016). Highlights update Dutch multidisciplinary guideline on schizophrenia in international perspective. PsyXpert, 7(1), 42–50.

Debbané, M., Salaminios, G., Luyten, P., Badoud, D., Armando, M., Solida Tozzi, A., Fonagy, P., & Brent, B. K. (2016). Attachment, neurobiology, and mentalizing along the psychosis continuum. Frontiers in Human Neuroscience, 10, 406.

Fonagy, P., Luyten, P., Allison, E., & Campbell, C. (2017). What we have changed our minds about: Borderline personality disorder as a limitation of resilience. Borderline Personality Disorder and Emotion Dysregulation, 4(1), 11.

Gumley, A., Taylor, H. E. F., Schwannauer, M., & MacBeth, A. (2014). A systematic review of attachment and psychosis: Measurement, construct validity and outcomes. Acta Psychiatrica Scandinavica, 129(4), 257–274.

Karterud, S. (2015). Mentalization-based group therapy (MBT-G): A theoretical, clinical, and research manual. Oxford University Press.

Salaminios, G., & Debbané, M. (2021). Associations between schizotypal personality features, mentalizing difficulties and thought problems in community adolescents. Early Intervention in Psychiatry, 15(3), 705–715.

Selten, J. P., van der Ven, E., Rutten, B. P., & Cantor-Graae, E. (2013). The social defeat hypothesis of schizophrenia: An update. Schizophrenia Bulletin, 39(6), 1180–1186.

Steenkamp, L., Weijers, J., Gerrmann, J., Eurelings-Bontekoe, E., & Selten, J. P. (2019). The relationship between childhood abuse and severity of psychosis is mediated by loneliness: An experience sampling study. Schizophrenia Research, 241, 306–311.

van den Berg, D. P., de Bont, P. A., van der Vleugel, B. M., de Roos, C., de Jongh, A., van Minnen, A., & van der Gaag, M. (2015). Prolonged exposure vs. eye movement desensitization and reprocessing for posttraumatic stress disorder in patients with a psychotic disorder: A randomized clinical trial. JAMA Psychiatry, 72(3), 259–267.

van der Lee, A., et al. (2016). Schizophrenia in the Netherlands: Continuity of care with first-episode and continuing treatment. BMC Psychiatry, 16, 393.

van Veldhuizen, J. R. (2007). FACT: A Dutch version of ACT. Community Mental Health Journal, 43(4), 421–433.

van Veldhuizen, J. R., & Bähler, M. (2013). Manual Flexible Assertive Community Treatment (FACT). Utrecht, The Netherlands: CCAF.

Varese, F., Smeets, F., Drukker, M., Lieverse, R., Lataster, T., Viechtbauer, W., Read, J., van Os, J., & Bentall, R. P. (2012). Childhood adversities increase the risk of psychosis: A meta-analysis. Schizophrenia Bulletin, 38(4), 661–671.

Weijers, J., ten Kate, C., Siecker, I., & Noij, Y. (2023). MBT voor mensen met een psychosegevoeligheid. Groepen, 18 (1), 23-40.

Weijers, J., ten Kate, C., Viechtbauer, W., Rampaart, L., Eurelings-Bontekoe, E., & Selten, J. (2021). Mentalization-based treatment for psychotic disorder: A rater-blinded, multi-center, randomized controlled trial. Psychological Medicine, 51(16), 2846–2855.

***Appendix II: Descriptives of Outcome Measures***

**Table 2**

**Outcome Descriptives by Treatment Condition**

| **Time** | **Group** | **Soc. Fun.** |  | **COM** | |  | **SOC** |  | **EMI** |  | **ToM** |  |  |
| --- | --- | --- | --- | --- | --- | --- | --- | --- | --- | --- | --- | --- | --- |
|  |  | ***M*** | ***SD*** | ***M*** | ***SD*** | | ***M*** | **SD** | **M** | **SD** | **M** | **SD** |  |
| T0 | TAU | 111.26 | 7.36 | 11.69 | | 0.87 | 11.53 | 2.10 | 10.75 | 2.98 | 16.14 | 2.85 |  |
|  | MBTp | 108.94 | 8.91 | 12.53 | | 1.81 | 11.59 | 2.40 | 10.06 | 2.99 | 17.45 | 2.11 |  |
| T1 | TAU | 111.67 | 6.58 | 11.31 | | 1.45 | 10.60 | 1.72 | 8.56 | 2.97 | 18.14 | 1.85 |  |
|  | MBTp | 113.28 | 7.82 | 12.00 | | 0.94 | 12.06 | 1.52 | 9.00 | 3.43 | 19.15 | 1.18 |  |
| T2 | TAU | 111.68 | 7.23 | 11.75 | | 0.93 | 10.80 | 1.61 | 8.56 | 2.37 | 17.71 | 2.37 |  |
|  | MBTp | 115.40 | 7.48 | 11.82 | | 0.95 | 12.94 | 2.82 | 9.41 | 2.58 | 19.05 | 1.61 |  |
| T3 | TAU | 110.39 | 6.30 | 11.13 | | 1.02 | 10.67 | 1.63 | 9.69 | 1.92 | 18.43 | 1.89 |  |
|  | MBTp | 111.87 | 6.64 | 11.76 | | 1.15 | 11.24 | 1.09 | 10.29 | 2.05 | 19.40 | 0.88 |  |

*Note.* *M* = mean; *SD* = standard deviation; T0 = Baseline, T1 = Post-treatment; T2 = 6-month follow-up; T3 = 5-year follow-up; Soc. Fun. = Social Functioning Scale; COM = Complexity of Representations; SOC is Understanding of social causality; EMI = Capacity for Emotional Investment; ToM = Theory of Mind. *Group* indicates participants randomized to TAU or MBTp.

**Appendix III: Statistical assumption tests**

*Social functioning (Social Functioning Scale):* We evaluated the statistical assumptions of the repeated-measures ANOVA for the primary outcome variable. Visual inspection of boxplots and spaghetti plots suggested that the data were approximately normally distributed. Q–Q plots of the residuals at each time point further indicated approximate normality. Skewness values ranged from –0.20 to 0.36 and kurtosis values from –0.33 to 0.03, both well within the commonly accepted range of –1 to +1, indicating no substantial deviations from normality.

Equality of error variances between the TAU and MBTp conditions was supported by Levene’s test at all time points, T1, p = .50; T2, p = .60; T3, p = .67; T4, p = .86. However, Mauchly’s test of sphericity was significant, p = .002, ε = .75, indicating a violation of the sphericity assumption; therefore, Huynh–Feldt corrected results are reported. The assumption of equality of covariance matrices was marginally met, Box’s M: *p* = .053. As a robustness check, results were compared across Pillai’s Trace and Wilks’ Lambda multivariate tests, which yielded identical conclusions.

*Understanding of social causality (Social Cognition and Object-Relations Scale):* We evaluated the statistical assumptions of the repeated-measures ANOVA for the SCORS-scale understanding of social causality. Visual inspection of boxplots and spaghetti plots suggested that the data were approximately roughly normally distributed at all timepoints. Q–Q plots of the residuals at each time point further indicated approximate normality. Skewness and kurtosis values of .41 and .81 respectively were within the commonly accepted range of –1 to +1, indicating no substantial deviations from normality.

Equality of error variances between the TAU and MBTp conditions was supported by Levene’s test at timepoints 1, 2, 3 and 4 (all p’s > .10). However, Mauchly’s test of sphericity was significant, p = .04, ε = .95, indicating a violation of the sphericity assumption; therefore, Huynh–Feldt corrected results will be used when referring to within-subjects results. The assumption of equality of covariance matrices was met, Box’s M (p = .53).

*Complexity of representations (Social Cognition and Object-Relations Scale):* We evaluated the statistical assumptions of the repeated-measures ANOVA for the SCORS-Scale Complexity of Representations. Visual inspection of boxplots and Q–Q plots of the residuals at each time point further indicated approximate normality. Skewness and kurtosis values of .41 and .80 respectively were within the commonly accepted range of –1 to +1, indicating no substantial deviations from normality.

Equality of error variances between the TAU and MBTp conditions was supported by Levene’s test at timepoints 1, 2, 3 and 4 (all p’s > .34). Mauchly’s test of sphericity was non-significant, all *p* = .12, indicating that the assumption held. Lastly, the assumption of equality of covariance matrices was met, Box’s M *(p* = .13)

*Capacity for emotional investment (Social Cognition and Object-Relations Scale):* We evaluated the statistical assumptions of the repeated-measures ANOVA for the primary outcome variable. Visual inspection of boxplots and spaghetti plots suggested that the data were approximately roughly normally distributed at all timepoints. Q–Q plots of the residuals at each time point further indicated approximate normality. Skewness and kurtosis values of .40 and .81 respectively were within the commonly accepted range of –1 to +1, indicating no substantial deviations from normality.

Equality of error variances between the TAU and MBTp conditions was supported by Levene’s test at timepoints 1, 2, 3 and 4 (all *p*’s > .73). Mauchly’s test of sphericity was nonsignificant, *p* = .18, indicating that the sphericity assumption held. The assumption of equality of covariance matrices was not me however, Box’s M (*p* = .03), indicating that Pillai’s Trace trace test results instead of Wilks’ Lambda should be used for interpretation. However since these yield identical conclusions, this assumption violation is of no consequence.

*Theory of Mind (Social Cognition and Object-Relations Scale):* We evaluated the statistical assumptions of the repeated-measures ANOVA for the primary outcome variable. Visual inspection of boxplots and spaghetti plots suggested that the data were approximately roughly normally distributed at all timepoints. Q–Q plots of the residuals at each time point further indicated approximate normality. Skewness and kurtosis values of .37 and .72 respectively were within the commonly accepted range of –1 to +1, indicating no substantial deviations from normality.

Mauchly’s test of sphericity was nonsignificant, p = .19, indicating that the sphericity assumption held. Equality of error variances between the TAU and MBTp conditions was supported by Levene’s test at timepoints 1, 2, (both p’s > .10), but not for T3 (p = .03) and T4 (p=.003), meaning that the assumption did not hold. The assumption of equality of covariance matrices was also not met, Box’s M (p = .01). We therefore conducted a secondary linear mixed method sensitivity analysis, for which the assumption of equality or error variances is not needed.

*Linear Mixed Model Sensitiviy Analyses:* Finally, we evaluated assumptions relevant to mixed-effects modeling, with particular attention to model fit, heteroscedasticity and multicollinearity. Random intercepts and random slopes for social functioning were specified at the participant level, with an unstructured variance–covariance matrix for the random effects. The inclusion of random slopes was considered essential to avoid inflated Type I error rates and overly narrow confidence intervals (Gurka, Edwards, & Muller, 2011; Schielzeth & Forstmeier, 2008). Model comparisons indicated that this specification provided the best fit relative to alternative covariance structures and random-effects specifications, as evidenced by the lowest information criteria values (AIC = 1712.07, BIC = 1690.66).

Heteroscedasticity was examined by regressing the squared standardized residuals on the predictors. The test was non-significant, R^2^=.009, p=.316, indicating that the assumption of homoscedasticity was met.

Multicollinearity among the fixed predictors was examined using tolerance and variance inflation factor (VIF) statistics obtained from an auxiliary linear regression model with time, treatment condition and their interaction term as indendent variables and social functioning as dependent variable. The tolerance values for treatment condition (.187), time (.496) and the time × treatment interaction (.157) corresponded to VIFs of 5.34, 2.02 and 6.42, respectively, indicating moderate collinearity. Because these values remained below the conventional VIF threshold of 10 (Field, 2018), multicollinearity was not considered severe. This degree of association is expected given the inclusion of an interaction term and does not compromise model estimation.

*References:*

Gurka, M. J., Edwards, L. J., & Muller, K. E. (2011). Avoiding bias in mixed model inference for fixed effects. *Statistics in Medicine*, 30(22), 2696–2707. https://doi.org/10.1002/sim.4293

Schielzeth, H., & Forstmeier, W. (2008). Conclusions beyond support: Overconfident estimates in mixed models. *Behavioral Ecology*, 20(2), 416–420. ttps://doi.org/10.1093/beheco/arn145

Field, A. P. (2018). *Discovering statistics using IBM SPSS Statistics* (5th ed.). SAGE Publications.

**Appendix IV: Participant Recruitment**

*Procedure:*

Our research assistant attempted to contact all 84 ex-participants that took part in the original study. No exceptions were made. If she was able to reach an ex-participant she explained we were going to do a follow-up study to see how participants were doing five years after the initial study. She also explained that this was for scientific purposes and that they would be reimbursed (€30,-) for their time. Any travel and parking expenses would also be paid for.

Each ex-participant was called. If a participant did not pick up his phone, a voicemail message was left. If the ex-participant had not responded to his voicemail within a week, or if his/her telephone number was out of order, an email was sent to the participant. For each ex-participant a maximum of five attempts were made to reach the participant by telephone and a maximum of three emails were sent. Fourteen ex-participants could not be reached because their telephone number was out of order and they did not respond to their email. Six participants did not pick up their phone and had email-addresses that were out of order. Nine ex-participants did not pick up their phone and did not respond to any email that was sent. Two ex-participants declined to participate and did not want to state a reason. Two participants declined because they were doing well. One participant declined because he was not doing well. Three participants declined because they were living too far away. One participant declined because he was afraid of contracting the corona virus.
